# Genome sequence of the *Ornithopus/Lupinus*-nodulating *Bradyrhizobium sp.* strain WSM471

**DOI:** 10.4056/sigs.4498256

**Published:** 2013-12-15

**Authors:** Wayne Reeve, Sofie De Meyer, Jason Terpolilli, Vanessa Melino, Julie Ardley, Rui Tian, Ravi Tiwari, John Howieson, Ronald Yates, Graham O’Hara, Mohamed Ninawi, Megan Lu, David Bruce, Chris Detter, Roxanne Tapia, Cliff Han, Chia-Lin Wei, Marcel Huntemann, James Han, I-Min Chen, Konstantinos Mavromatis, Victor Markowitz, Natalia Ivanova, Ioanna Pagani, Amrita Pati, Lynne Goodwin, Tanja Woyke, Nikos Kyrpides

**Affiliations:** 1 Centre for Rhizobium Studies, Murdoch University, Western Australia, Australia; 2 Department of Agriculture and Food, Western Australia, Australia; 3 DOE Joint Genome Institute, Walnut Creek, California, USA; 4 Los Alamos National Laboratory, Bioscience Division, Los Alamos, New Mexico, USA; 5 Biological Data Management and Technology Center, Lawrence Berkeley National Laboratory, Berkeley, California, USA

**Keywords:** root-nodule bacteria, nitrogen fixation, rhizobia, *Alphaproteobacteria*

## Abstract

*Bradyrhizobium sp.* strain WSM471 is an aerobic, motile, Gram-negative, non-spore-forming rod that was isolated from an effective nitrogen- (N_2_) fixing root nodule formed on the annual legume *Ornithopus pinnatus* (Miller) Druce growing at Oyster Harbour, Albany district, Western Australia in 1982. This strain is in commercial production as an inoculant for *Lupinus* and *Ornithopus*. Here we describe the features of *Bradyrhizobium sp.* strain WSM471, together with genome sequence information and annotation. The 7,784,016 bp high-quality-draft genome is arranged in 1 scaffold of 2 contigs, contains 7,372 protein-coding genes and 58 RNA-only encoding genes, and is one of 20 rhizobial genomes sequenced as part of the DOE Joint Genome Institute 2010 Community Sequencing Program.

## Introduction

The most abundant form of nitrogen (N) occurs in the atmosphere as a chemically inert dinitrogen (N_2_) gas. However, N_2_ needs to be converted first into a biologically useable form through the unique process of N_2_ fixation [1]. The incorporation of fixed N into biologically essential macromolecules provides the basis for the continuance of life on Earth. Bioavailable N can be chemically synthesized (primarily through the products obtained from the Haber-Bosch process) or biologically fixed by N_2_-fixing diazotrophs. The highest contribution to biological fixation occurs from the process of symbiotic nitrogen fixation (SNF). The estimated total annual input from SNF ranges from 139 - 175 million tons [2] which provides ~70% of the N currently utilized in agriculture. However, various constraints from edaphic conditions can limit SNF capacity in certain agricultural areas. To extend productive crops and pastures into these regions, considerable efforts have been devoted to sourcing legume hosts and their compatible microsymbionts from different geographical locations that are edaphically and climatically suited to the challenging areas into which they are to be introduced [3].

These selection programs have enabled the domestication of new Mediterranean legume species that have overcome the deficiencies of the use of traditional species [4]. Seven species new to Australian agriculture have been commercialized since 1993 including the Papilionoid legume *Ornithopus sativus* (serradella) [4]. This hard-seeded deep-rooted and acid tolerant pasture legume has shown particular promise in acidic sandy soils exposed to low rainfall [4], with the potential to be established in four million hectares of sandy soils for which no other suitable legume pasture exists [5]. The hard seeded nature of this legume makes it well adapted to crop rotation systems [4]. Currently, serradella is the most widely sown pasture in Western Australia and has proven to be a highly productive legume with high nutritive value [4].

The strains of lupin-nodulating *Bradyrhizobium* that also nodulate seradella are unusual since they have the capacity to establish symbioses with Mediterranean derived herbaceous and crop legumes endemic to the cool climatic regions of the world. Before the 1990s, the commercial inoculant for serradella (*Ornithopus* spp.) in Australia was *Bradyrhizobium sp.* strain WU425, however during the breeding and evaluation of well adapted cultivars of *O. sativus*, it was revealed that WSM471 produced 15% more biomass with this legume than did WU425 [5]. Strain WSM471 was isolated from nodules of *O. pinnatus* collected in Western Australia, in 1982, although it was almost certainly accidentally introduced to Australia [6]. Because of its superior capacity to fix nitrogen with *O. sativus* relative to other strains of *Bradyrhizobium*, strain WSM471 was released as a commercial inoculant for this legume in Australia in 1996 [7] and remains in current usage. This strain is also the commercial “back-up” for inoculation of lupins in Australia. Here we present a summary classification and a set of general features for *Bradyrhizobium sp.* strain WSM471 together with the description of the complete genome sequence and its annotation.

## Classification and general features

**Figure 1:**
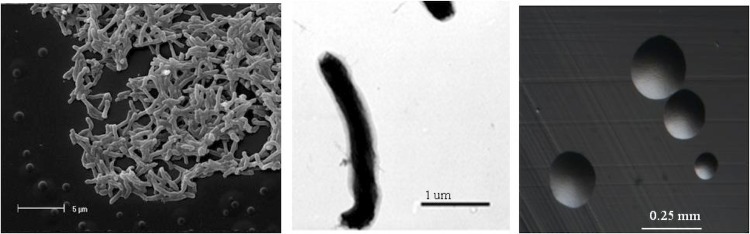
Images of *Bradyrhizobium sp.* strain WSM471 using scanning (Left) and transmission (Center) electron microscopy as well as light microscopy to visualize colony morphology on a solid medium (Right).

*Bradyrhizobium sp.* strain WSM471 is a motile, Gram-negative, non-spore-forming rod (Figure 1 Left, Center) in the order *Rhizobiales* of the class *Alphaproteobacteria*. It is slow growing, forming colonies within 7-10 days when grown on half Lupin Agar (½LA) [8] at 28°C. Colonies on ½LA are white-opaque, slightly domed, moderately mucoid with smooth margins (Figure 1 Right).

**Figure 2:**
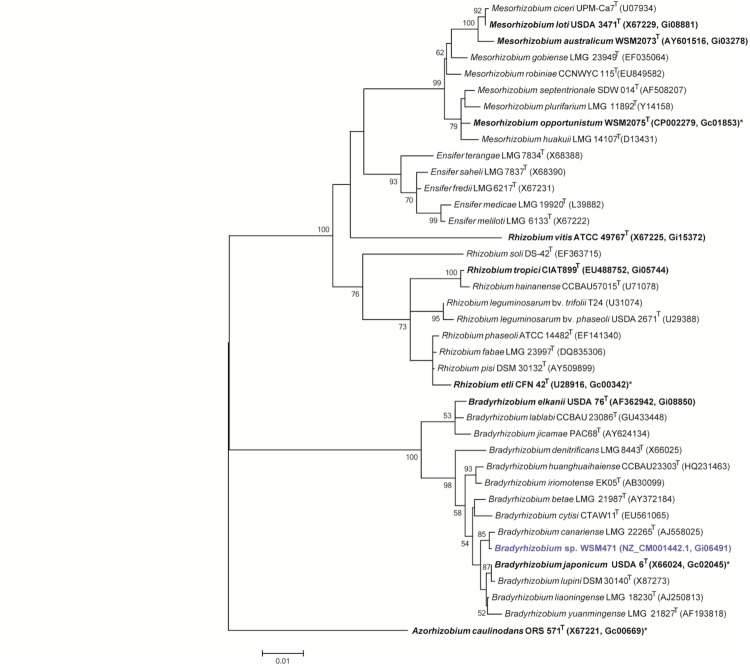
Phylogenetic tree showing the relationships of *Bradyrhizobium sp.* strain WSM471 (shown in blue print) with some of the root nodule bacteria in the order *Rhizobiales* based on aligned sequences of the 16S rRNA gene (1,310 bp internal region). All sites were informative and there were no gap-containing sites. Phylogenetic analyses were performed using MEGA, version 5.05 [19]. The tree was built using the maximum likelihood method with the General Time Reversible model. Bootstrap analysis [20] with 500 replicates was performed to assess the support of the clusters. Type strains are indicated with a superscript T. Strains with a genome sequencing project registered in GOLD [21] are in bold print and the GOLD ID is mentioned after the accession number. Published genomes are designated with an asterisk.

Minimum Information about the Genome Sequence (MIGS) is provided in Table 1. Figure 2 shows the phylogenetic relationship of *Bradyrhizobium sp.* strain WSM471 in a 16S rRNA sequence based tree. This strain clusters closest to *Bradyrhizobium canariense* LMG 22265^T^ and *Bradyrhizobium japonicum* LMG 6138^T^ with 99.9% and 99.5% sequence identity, respectively.

**Table 1:** Classification and general features of *Bradyrhizobium sp.* strain WSM471 according to the MIGS recommendations [9].

**MIGS ID**	**Property**	**Term**	**Evidence code**
	Current classification	Domain *Bacteria*	TAS [10]
		Phylum *Proteobacteria*	TAS [11]
		Class *Alphaproteobacteria*	TAS [12,13]
		Order *Rhizobiales*	TAS [13,14]
		Family *Bradyrhizobiaceae*	TAS [13,15]
		Genus *Bradyrhizobium*	TAS [16]
		Species *Bradyrhizobium sp.*	IDA
	
	Gram stain	Negative	TAS [16]
	Cell shape	Rod	TAS [16]
	Motility	Motile	TAS [16]
	Sporulation	Non-sporulating	TAS [16]
	Temperature range	Mesophile	TAS [16]
	Optimum temperature	28°C	TAS [16]
	Salinity	Not reported	
MIGS-22	Oxygen requirement	Aerobic	TAS [16]
	Carbon source	Varied	TAS [16]
	Energy source	Chemoorganotroph	TAS [16]
MIGS-6	Habitat	Soil, root nodule on host	IDA
MIGS-15	Biotic relationship	Free living, symbiotic	IDA
MIGS-14	Pathogenicity	Non-pathogenic	NAS
	Biosafety level	1	TAS [17]
	Isolation	Root nodule	IDA
MIGS-4	Geographic location	Albany, Western Australia	IDA
MIGS-5	Nodule collection date	1982	IDA
MIGS-4.1	Longitude	117.96	IDA
MIGS-4.2	Latitude	-34.98	IDA
MIGS-4.3	Depth	Not recorded	
MIGS-4.4	Altitude	69m	IDA

Evidence codes – IDA: Inferred from Direct Assay; TAS: Traceable Author Statement (i.e., a direct report exists in the literature); NAS: Non-traceable Author Statement (i.e., not directly observed for the living, isolated sample, but based on a generally accepted property for the species, or anecdotal evidence). These evidence codes are from the Gene Ontology project [18].

### Symbiotaxonomy

*Bradyrhizobium sp.* strain WSM471 was isolated from nodules of *Ornithopus pinnatus* collected from Oyster Harbour, near Albany, Western Australia (34.98 lat; 117.96 long), in 1982. The purpose of the collection of the nodules that gave rise to WSM471 was to seek strains of nodulating bacteria that might improve the winter nitrogen fixation capacity of the symbiosis with *Lupinus angustifolius*. This symbiosis seemed to be limited by low winter temperatures, which was later confirmed by Peltzer *et al.* [22]. Strain WSM471 is highly effective for nitrogen fixation with the grain legumes *L. pilosus, L. angustifolius* and *L. atlanticus*, and also the forage legumes *O. pinnatus, O. sativus* and *O. compressus* [5,23]. Because WSM471 has a broad range for symbiotic nitrogen fixation across both pulse and forage legumes, and is in commercial usage, it was chosen as a candidate strain for sequencing.

## Genome sequencing and annotation information

### Genome project history

This organism was selected for sequencing on the basis of its environmental and agricultural relevance to issues in global carbon cycling, alternative energy production, and biogeochemical importance, and is part of the Community Sequencing Program at the U.S. Department of Energy, Joint Genome Institute (JGI) for projects of relevance to agency missions. The genome project is deposited in the Genomes OnLine Database [21] and an improved-high-quality-draft genome sequence in IMG. Sequencing, finishing and annotation were performed by the JGI. A summary of the project information is shown in Table 2.

**Table 2:** Genome sequencing project information for *Bradyrhizobium sp.* strain WSM471.

**MIGS ID**	**Property**	**Term**
MIGS-31	Finishing quality	Non-contiguous Finished
MIGS-28	Libraries used	Illumina GAii shotgun and paired end 454 libraries
MIGS-29	Sequencing platforms	Illumina GAii and 454 GS FLX Titanium technologies
MIGS-31.2	Sequencing coverage	6.9× 454 paired end, Illumina 625.6
MIGS-30	Assemblers	Velvet1.0.13, Newbler 2.3, phrap 4.24
MIGS-32	Gene calling methods	Prodigal 1.4, GenePRIMP
	Genbank ID	CM001442
	Genbank Date of Release	February 2, 2012
	GOLD ID	Gi06491
	NCBI project ID	61807
	Database: IMG	2508501009
	Project relevance	Symbiotic N_2_-fixation, agriculture

### Growth conditions and DNA isolation

*Bradyrhizobium sp.* strain WSM471 was grown to mid logarithmic phase in TY rich medium [24] on a gyratory shaker at 28°C. DNA was isolated from 60 mL of cells using a CTAB (Cetyl trimethyl ammonium bromide) bacterial genomic DNA isolation method [25].

### Genome sequencing and assembly

The genome of *Bradyrhizobium sp.* WSM471 was generated at the DOE Joint Genome Institute (JGI) using a combination of Illumina [26] and 454 technologies [27]. An Illumina GAii shotgun library which generated 67,039,982 reads totaling 5,095 Mb and 1 paired end 454 library with an average insert size of 5 Kb which generated 397,976 reads totaling 83.7 Mb of 454 were generated for this genome. All general aspects of library construction and sequencing performed at the JGI can be found at the JGI website [25]. The initial draft assembly contained 236 contigs in 2 scaffolds. The 454 Titanium standard data and the 454 paired end data were assembled together with Newbler, version 2.3. The Newbler consensus sequences were computationally shredded into 2 Kb overlapping fake reads (shreds). Illumina sequencing data was assembled with Velvet, version 1.0.13 [28], and the consensus sequence were computationally shredded into 1.5 kb overlapping fake reads (shreds). We integrated the 454 Newbler consensus shreds, the Illumina Velvet consensus shreds and the read pairs in the 454 paired end library using parallel phrap, version SPS - 4.24 (High Performance Software, LLC). The software Consed [29-31] was used in the following finishing process. Illumina data was used to correct potential base errors and increase consensus quality using the software Polisher developed at JGI (Alla Lapidus, unpublished). Possible mis-assemblies were corrected using gapResolution (Cliff Han, unpublished), Dupfinisher [32], or sequencing cloned bridging PCR fragments with subcloning. Gaps between contigs were closed by editing in Consed, by PCR and by Bubble PCR (J-F Cheng, unpublished) primer walks. A total of 327 additional reactions were necessary to close gaps and to raise the quality of the finished sequence. The estimated genome size is 7.8 Mb and the final assembly is based on 53.8 Mb of 454 draft data which provides an average 6.9× coverage of the genome and 4,879.9 Mb of Illumina draft data which provides an average 625.6× coverage of the genome.

### Genome annotation

Genes were identified using Prodigal [33] as part of the DOE-JGI Annotation pipeline [34] followed by a round of manual curation using the JGI GenePRIMP pipeline [35]. The predicted CDSs were translated and used to search the National Center for Biotechnology Information (NCBI) non-redundant database, UniProt, TIGRFam, Pfam, PRIAM, KEGG, COG, and InterPro databases. These data sources were combined to assert a product description for each predicted protein. Non-coding genes and miscellaneous features were predicted using tRNAscan-SE [36], RNAMMer [37], Rfam [38], TMHMM [39], and SignalP [40]. Additional gene prediction analyses and functional annotation were performed within the Integrated Microbial Genomes (IMG-ER) platform [41].

## Genome properties

The genome is 7,784,016 nucleotides with 63.40% GC content (Table 3) and comprised of 1 scaffold (Figure 3a, Figure 3b) of 2 contigs. From a total of 7430 genes, 7,372 were protein encoding and 58 RNA only encoding genes. Within the genome, 274 pseudogenes were also identified. The majority of genes (74.10%) were assigned a putative function whilst the remaining genes were annotated as hypothetical. The distribution of genes into COGs functional categories is presented in Table 4.

**Table 3:** Genome Statistics for *Bradyrhizobium sp.* strain WSM471.

**Attribute**	**Value**	**% of Total**
Genome size (bp)	7,784,016	100.00
DNA coding region (bp)	6,519,740	83.76
DNA G+C content (bp)	4,935,436	63.40
Number of scaffolds	1	
Number of contigs	2	
Total genes	7,430	100.00
RNA genes	58	0.78
rRNA operons	1	0.01
Protein-coding genes	7,372	99.22
Genes with function prediction	5,506	74.10
Genes assigned to COGs	5,507	74.12
Genes assigned Pfam domains	5,758	77.50
Genes with signal peptides	834	11.22
Genes with transmembrane helices	1,739	23.41
CRISPR repeats	0	

**Figure 3a:**
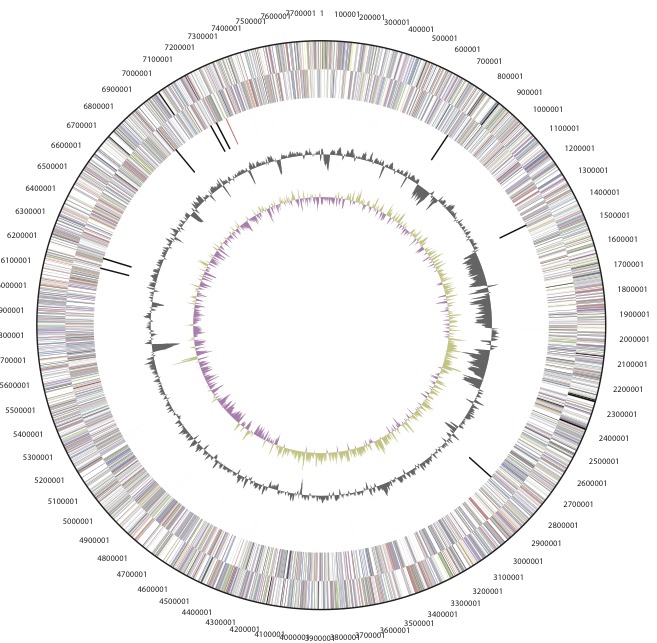
Graphical circular map of the chromosome of *Bradyrhizobium sp.* strain WSM471. From outside to the center: Genes on forward strand (color by COG categories as denoted by the IMG platform), Genes on reverse strand (color by COG categories), RNA genes (tRNAs green, sRNAs red, other RNAs black), GC content, GC skew.

**Figure 3b:**
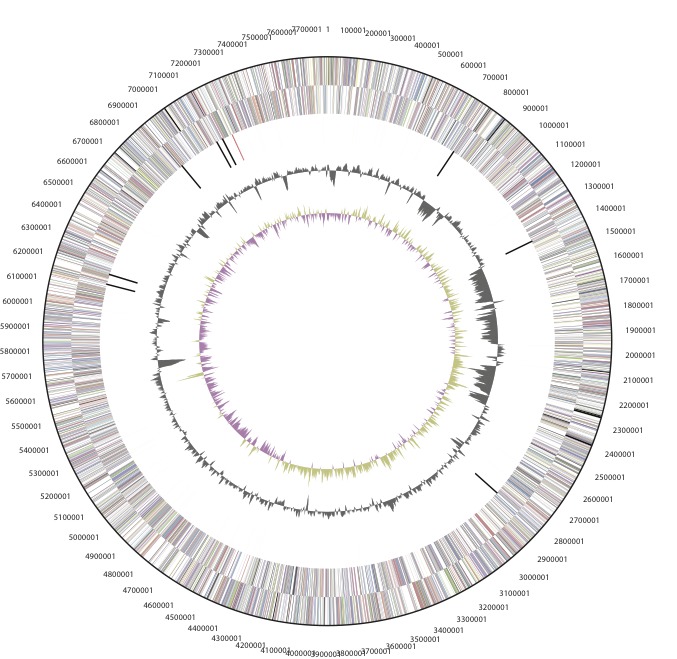
Graphical circular map of the plasmid of *Bradyrhizobium sp.* strain WSM471. From outside to the center: Genes on forward strand (color by COG categories as denoted by the IMG platform), Genes on reverse strand (color by COG categories), RNA genes (tRNAs green, sRNAs red, other RNAs black), GC content, GC skew.

**Table 4:** Number of protein coding genes of *Bradyrhizobium sp.* strain WSM471 associated with the general COG functional categories.

**Code**	**Value**	**%age**	**Description**	
J	208	3.37	Translation, ribosomal structure and biogenesis
A	1	0.02	RNA processing and modification
K	395	6.41	Transcription
L	268	4.35	Replication, recombination and repair
B	2	0.03	Chromatin structure and dynamics
D	33	0.54	Cell cycle control, mitosis and meiosis
Y	0	0.00	Nuclear structure
V	85	1.38	Defense mechanisms
T	369	5.98	Signal transduction mechanisms
M	327	5.30	Cell wall/membrane biogenesis
N	121	1.96	Cell motility
Z	1	0.02	Cytoskeleton
W	0	0.00	Extracellular structures
U	102	1.65	Intracellular trafficking and secretion
O	191	3.10	Posttranslational modification, protein turnover, chaperones
C	410	6.65	Energy production conversion
G	406	6.58	Carbohydrate transport and metabolism
E	645	10.46	Amino acid transport metabolism
F	88	1.43	Nucleotide transport and metabolism
H	234	3.79	Coenzyme transport and metabolism
I	335	5.43	Lipid transport and metabolism
P	304	4.93	Inorganic ion transport and metabolism
Q	238	3.86	Secondary metabolite biosynthesis, transport and catabolism
R	770	12.49	General function prediction only
S	634	10.28	Function unknown
-	1,923	25.88	Not in COGS
